# Human amniotic epithelial cells inhibit granulosa cell apoptosis induced by chemotherapy and restore the fertility

**DOI:** 10.1186/s13287-015-0148-4

**Published:** 2015-08-25

**Authors:** Qiuwan Zhang, Minhua Xu, Xiaofen Yao, Ting Li, Qian Wang, Dongmei Lai

**Affiliations:** International Peace Maternity and Child Health Hospital, School of Medicine, Shanghai Jiao Tong University, 145, Guang-Yuan Road, Shanghai, 200030 People’s Republic of China; Institute of Embryo-Fetal Original Adult Disease Affiliated to Shanghai Jiao Tong University School of Medicine, Shanghai, 200030 People’s Republic of China

## Abstract

**Introduction:**

Premature ovarian failure and insufficiency are significant long-term side-effects of chemotherapy for female cancer patients. Recently, stem cell transplantation has been identified as a promising treatment for premature ovarian failure and insufficiency. We have previously demonstrated that human amniotic epithelial cells (hAECs) migrate into injured tissue and promote the recovery of ovarian function in chemoablated mice. However, the molecular mechanism guiding this process remains unclear.

**Methods:**

To further investigate the effect of hAECs on chemotherapy-induced apoptosis, cultured primary hAECs were injected intravenously into mice treated with cyclophosphamide and busulphan. Apoptosis of granulosa cells was observed by TUNEL staining, and apoptosis-related gene expression was performed on ovarian tissue by real-time PCR and Western blot 7 days after hAEC transplantation. Additionally, the ovarian function and fertility of mice were assessed via counts of follicles and mating experiments at 4 weeks after hAEC transplantation.

**Results:**

hAECs significantly inhibited tumor necrosis factor-alpha-mediated granulosa cell apoptosis induced by chemotherapeutics and reduced the inflammatory reaction in ovaries at 7 days after transplantation. In addition, 4 weeks after transplantation, hAECs promoted the development of follicles and increased the number of cumulus oocyte complexes in chemoablated mice. Furthermore, hAECs improved ovarian mass and increased the number of follicles compared to those of the chemoablated group, and hAEC transplantation partially rescued the fertility of chemoablated mice.

**Conclusions:**

hAEC transplantation promotes ovarian function by inhibiting tumor necrosis factor-alpha-mediated cell apoptosis and reducing inflammation in chemotherapy-induced premature ovarian failure. These results suggest a potential molecular mechanism for the effective therapy of hAEC transplantation in chemotherapy-induced premature ovarian failure and insufficiency.

**Electronic supplementary material:**

The online version of this article (doi:10.1186/s13287-015-0148-4) contains supplementary material, which is available to authorized users.

## Introduction

Chemotherapy is commonly used to treat both malignant neoplasms and disorders of the immune system, and its use is accompanied by a host of side-effects. For women in particular, the use of chemotherapy can lead to irreversible premature ovarian failure and insufficiency (POF/POI). Additionally, POF/POI is the main cause of female infertility [1], and the risk of POF/POI varies with age, occurring in up to 1 % of women between the ages of 30 and 40 years [2]. Women with POF/POI suffer from menopausal symptoms including hot flushes, vaginal dryness and increased risk of developing osteoporosis, all of which diminish the patient's quality of life, especially nonpregnant women [3].

Currently, there is no effective treatment for POF/POI, and medical reversal can be challenging. Studies have demonstrated that ovaries in the reproductive stage contain a small amount of active mitotic germ cells, with stem cell properties and the ability to differentiate into oocytes [4]. Stem cells have indefinite proliferative capacity and multi-directional differentiation potential, and hold great promise in tissue engineering for regenerative medicine. However, clinical applications of stem cells are quite limited at the present time, partly due to the low quantities available for use and research.

Encouraging reports have revealed that stem cells derived from a variety of human tissues including skin-derived mesenchymal stem cells [5], adipose-derived stem cells [6] and human amniotic fluid stem cells [7, 8] could rescue chemotherapy-induced ovarian injury. Human amniotic epithelial cells (hAECs) derived from term placentas demonstrate key advantages over other stem cell lineages, particularly their nonimmunogenic and nontumorigenic characteristics [9]. Furthermore, these cells can be readily harvested in ways that avoid the ethical issues that have arisen from other sources. Evidence has shown that cultured hAECs have stem cell properties, lose telomerase activity, and have the potential to differentiate into three embryonic germ layers [10, 11]. Various studies have highlighted the utility of hAECs as a novel and viable source of stem cells for transplantation in tissue injuries, such as reducing proliferation and activation of primary lung fibroblasts in mice as well as reducing hyperoxia-induced lung inflammation and structural lung damage [12, 13]. hAECs may promote lung repair by directly modulating macrophage recruitment and polarization [14], reducing fetal brain injuries that may occur in response to intrauterine inflammation [15]. Interestingly, however, preterm hAECs exerted substantially less protective effects than full-term hAECs when administered following acute lung injury [16]. A previous study found that hAECs cultured in medium containing serum substitute supplement (SSS) could differentiate into cells expressing specific germ cell markers [17]. Previously, we demonstrated that transplantation of term hAECs could lead them to migrate into injured ovarian tissue, differentiate into granulosa cells (GCs) and promote the recovery of ovarian function [18]. Thus, hAECs may have the potential to treat POF/POI; however, the specific cellular and molecular mechanisms guiding this process have remained unclear.

In order to further characterize the effect of hAECs on chemotherapy-induced ovarian injury, we established an animal model of POF/POI by intraperitoneal injection with cyclophosphamide combined with busulfan [18]. Cultured hAECs were transplanted into chemoablated mice by tail vein injection, and the long-term recovery of ovarian function was evaluated. Additionally, we evaluated apoptosis and inflammation induced by chemotherapy. We also offer a characterization and discussion on the protective role of hAECs on the development process of follicle and ovarian function.

## Materials and methods

### Isolation and culture of hAECs

Human placentas were obtained at term pregnancy during uncomplicated Caesarean sections with written and informed consent from woman who tested negative for HIV-I, and hepatitis B and C. This work has been approved by the Institutional Ethics Committee of the International Peace Maternity and Child Health Hospital, and written informed consent was obtained from all participants. Amniotic membranes were mechanically separated from the chorion of the placenta and dissected into several segments, following pre-cool phosphate-buffered saline (PBS) washing. The membrane segments were digested with 0.25 % trypsin/EDTA at 37 °C for 25 min. The resulting cell suspensions were seeded in 100 mm cell culture plates containing DMEM/F12 (Gibco, Grand Island, NY, USA) medium supplemented with 10 % fetal bovine serum (FBS; Gibco), streptomycin (100 U/mL; Gibco), and penicillin (100 U/Ml; Gibco), and incubated at 37 °C in an incubator containing 5 % CO_2_. Once the density of cells reached 80–90 % confluency, cells were collected for subsequent experiments.

### POF/POI model establishment

A total of 97 female C57/BL6 wild-type mice aged from 7 to 8 weeks were obtained from Shanghai Experimental Animal Center of Chinese Academy of Sciences. Mice sorted into the chemoablated group (Cy; n = 44) were given a single intraperitoneal injection of busulfan (30 mg/kg; Sigma) and cyclophosphamide (120 mg/kg, Hengrui, Jiangsu, China) to induce premature ovarian failure, as previously described [5]. Mice in the sham-control group (Sham) were injected with an equal volume of PBS (n = 27). Mice in the hAEC-treated group (Cy + hAECs; n = 26) were injected with hAECs (2 × 10^6^ in PBS) in the tail vein at 7 days post-induction. The body weight for each mouse was recorded.

All procedures for animals were approved by the Institutional Animal Care and Use Committee of Shanghai, and were performed in accordance with the National Research Council Guide for Care and Use of Laboratory Animals. Efforts were made to minimize animal suffering and limit the number of animals used in the study.

### RNA extraction and real-time quantitative PCR analysis

Total RNA was isolated from cultured hAECs and ovaries of mice by using Trizol (Invitrogen, Carlsbad, CA, USA) using previously described methods [18]. Real-time quantitative PCR reactions were performed in triplicate, using the SYBR Green Real-time PCR Master Mix (Applied, Takara, Japan). PCR primers were designed according to cDNA sequences in the NCBI database. Primer sequences used for real-time quantitative PCR are listed in Additional file 1: Table S1. Cycling conditions for the PCR machine were as follows: 95 °C 5 mins, 60 °C 34 s for 35 cycles. Gene expression levels were evaluated using the delta-delta CT method, standardized to levels of GAPDH amplification.

### Histologic evaluation

**Fig. 1:**
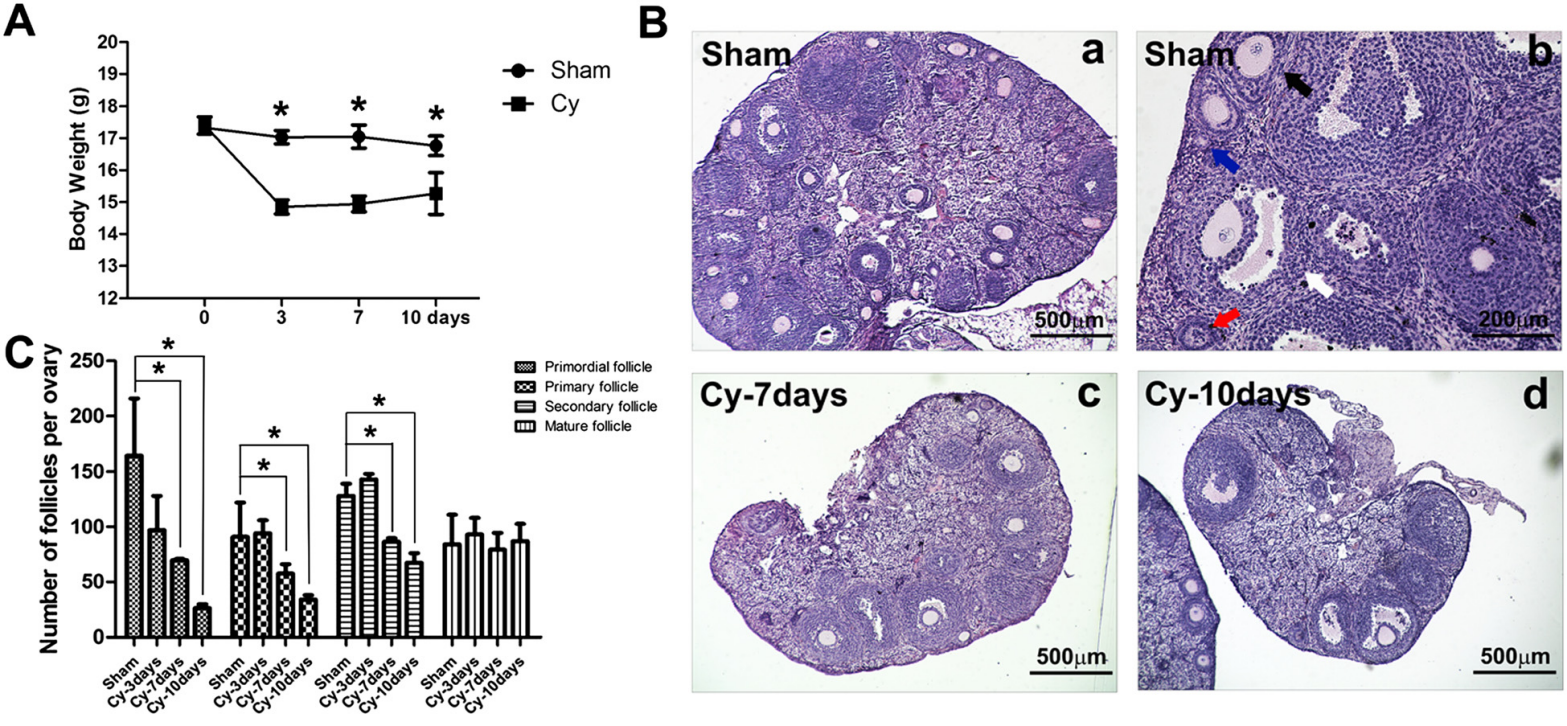
Chemotherapy reduced body weight of mice and the number of ovarian follicles. **A** Bar graph illustrating the body weight of mice in the sham and chemoablated (Cy) groups. **B** H&E staining of ovaries in sham and treatment groups, 7 and 10 days post-induction. Blue arrow indicated primordial follicle; red arrow indicated primary follicle; black arrow indicated secondary follicle; white arrow indicated mature follicle in B-b. **C** Bar graph representing the number of follicles at different stages of development. Data represent means ± SEM; *p < 0.05 versus Sham. Scale bar = 500 μm (*a, c* and *d*), 200 μm in *b. Sham* sham group (n = 6), *Cy-3days* chemoablated 3-day group (n = 6), *Cy-7days* chemoablated 7-day group (n = 6), *Cy-10days* chemoablated 10-day group (n = 6)

Ovaries were collected from sham and chemoablated groups at 3, 7, and 10 days after chemotherapy, and at 7 and 28 days after hAEC transplantation. Ovaries were fixed in Bouin's solution (containing 5 % acetic acid, 9 % formaldehyde and 0.9 % picric acid), paraffin-embedded and serially sectioned at a thickness of 5 μm. Hematoxylin-eosin (H&E) staining was used to evaluate the morphological structure of the ovaries, which was evaluated using light microscopy. Follicles were categorized and counted in every fifth section of the ovary, in a method as previously described [19, 20]. Briefly, a primordial follicle was defined as GCs surrounding a single fusiform oocyte. A primary follicle was surrounded by at least three granule cells, resulting in a cubic shape, and a secondary follicle appeared surrounded by at least two layers of GCs with no follicular cavity. Mature follicles (Antral follicles) contain at least two GCs and demonstrated evidence of follicular cavity (Fig. 1B-*b*).

### TUNEL assay

In situ Cell Death Detection Kit (Roche, Germany) was used to detect apoptosis in the ovarian tissues of mice according to manufacturer’s instructions. The nucleus was stained with DAPI, and the ovarian sections were evaluated using fluorescence microscopy (Leica, Germany).

### Western blot analysis

For analysis of Western blot, protein lysate from fresh ovarian tissue was prepared, separated on 8 % SDS-polyacrylamide gel and transferred to a polyvinyldifluoridine (PVDF) membrane (Millipore). Membranes were blocked with 10 % non-fat milk in Tris–HCl (10 mM, pH 7.4) containing 150 mM NaCl, and 1 % Nonidet P-40 and separately incubated with the following specific antibodies at 4 °C overnight: rabbit polyclonal caspase-3 antibody (1:500 dilution; CST) and rabbit monoclonal actin antibody (1:10000; Abcam, Cambridge, UK,). After washing, membranes were incubated with horseradish peroxidase-conjugated goat anti-rabbit IgG (1:1000; Abcam). Visualization of blots was performed using a standard protocol for ECL (Santa Cruz Biotechnology, Santa Cruz, CA, USA). The relative intensity of protein bands was quantified by digital densitometry (Image J software, National Institutes of Health, USA). Actin levels were used as internal standards.

### Mouse superovulation

Three groups of female C57/BL6 mice were superovulated at 1 month after hAEC transplantation via a single intraperitoneal injection of pregnant mare serum gonadotropin (PMSG; 10 UI), followed by injection of human chorionic gonadotropin (hCG; 10 UI) 48 h later. The cumulus oocyte complexes (COC) were collected from the ampulla portion of the oviduct at 14–16 h after hCG injection. Superovulated ovary tissues were fixed in Bouin's solution for further H&E staining as described above.

### Mating protocol

Female mice underwent intraperitoneal injections with busulfan (30 mg/kg) and cyclophosphamide (120 mg/kg), and hAECs were transplanted into POF mice at 7 days post-induction. Four weeks after hAEC transplantation, two female mice in each group were enclosed with an untreated male for 1 month. Once mating was confirmed by formation of the fertilization plugs, the females were separated prior to birth of the litter. The number of pups per pregnancy was counted.

### Statistical analysis

The mean and standard error of the mean (SEM) were calculated for experimental variables. Statistical significance was calculated using GraphPad Prism (GraphPad Software Inc., San Diego, CA, USA). Body weights and ovarian weights were analyzed using the repeated measures analysis of variance (ANOVA) with the Bonferroni post-hoc test. Western blot, quantitative PCR and follicular counting data were analyzed using two-way ANOVA testing with the least significant difference (LSD) test. Confidence intervals of 95 % were deemed statistically significant. Differences between groups were considered significant when *p* < 0.05.

## Results

### Chemotherapy induces the loss of ovarian follicles

Chemotherapeutics cause a series of secondary effects to the immune and/or reproductive systems. The sensitivity of the ovary to chemotherapeutics is gradually increased from puberty to adulthood [21]. In the present study, we evaluated the body weight of mice following chemotherapy and observed a significant post-chemotherapy reduction that was most pronounced at 3 and 7 days (Fig. 1A, *p* < 0.05). To observe the specific changes to ovarian structure, the ovaries of mice in the sham group and chemoablated group were collected for pathological analysis. The ovaries of mice in the chemoablated group were atrophic compared to controls, containing fewer developing follicles at 7 and 10 days after chemotherapy (Fig. 1B). Additionally, we analyzed the number of follicles over the course of treatments. At 7 and 10 days following chemotherapy, chemoablated mice demonstrated a statistically significant reduction in the number of primordial, primary and secondary ovarian follicles (Fig. 1C, *p* < 0.05).

### Chemotherapy-induced apoptosis in ovarian granulosa cells occurs mainly in secondary follicles

**Fig. 2:**
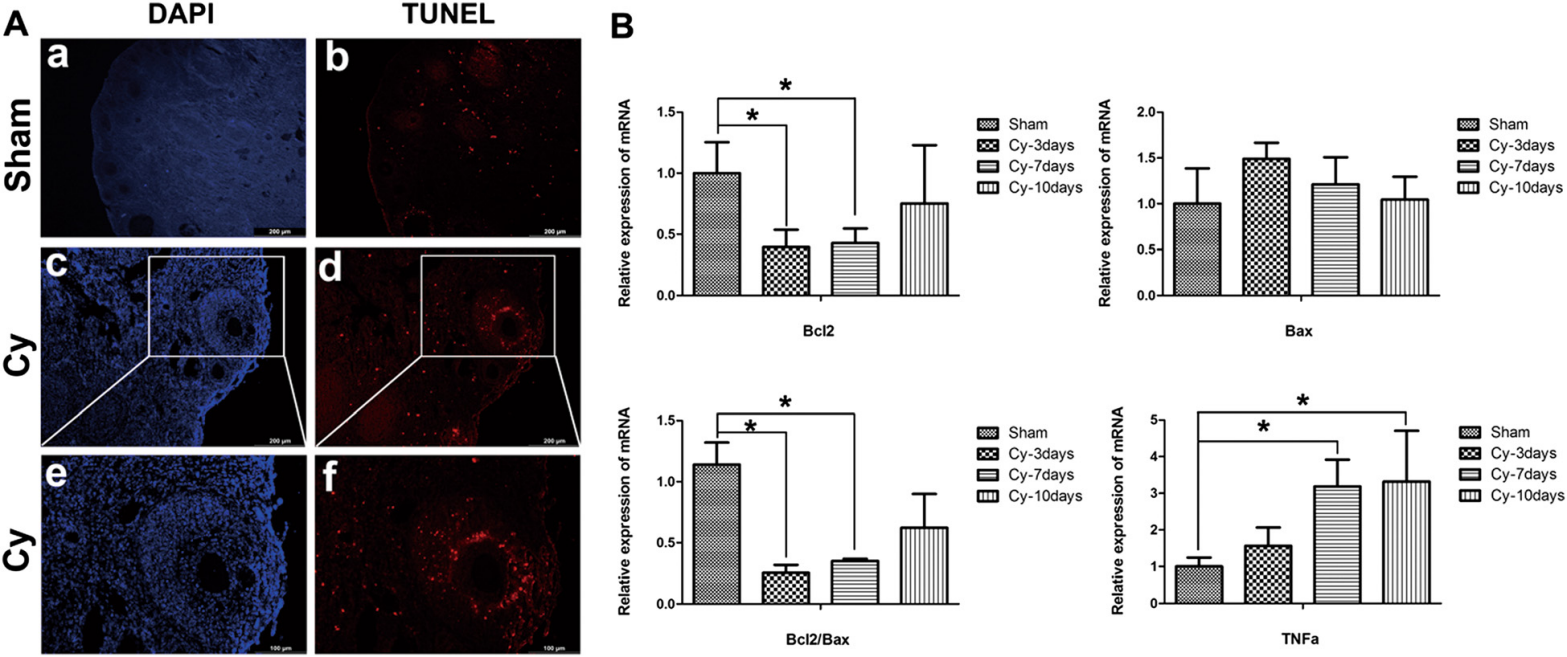
Chemotherapy-induced apoptosis of granulosa cells within developing follicle. **A** TUNEL-positive cells were observed in the ovary tissue at 7 days after chemotherapy. The red stain indicates TUNEL-positive granulosa cells. The blue DAPI stain indicates the cell nucleus. **B** Relative expression of Bcl2, Bax and TNF-α mRNA in ovarian tissue at 3, 7 and 10 days after chemotherapy. Data represent means ± SEM; *p < 0.05 versus Sham. Scale bar = 200 μm (*a, b, c* and *d*), 100 μm (*e* and *f*). *Sham* sham group (n = 6), *Cy-3days* chemoablated 3-day group (n = 6), *Cy-7days* chemoablated 7-day group (n = 6), *Cy-10days* chemoablated 10-day group (n = 6)

To interrogate the mechanism contributing to chemotherapy-induced loss of follicles, we investigated the effects of chemoablation on cell apoptosis in ovarian tissue. TUNEL-positive cells were observed in the ovarian section of mice at 7 days after chemotherapy, suggesting apoptosis was largely restricted to the GC layer of secondary follicle, in proximity to the oocytes (Fig. 2A). To this effect, we observed significant downregulation of the antiapoptotic gene Bcl2 in ovaries of chemoablated mice (Fig. 2B, *p* < 0.05). Studies have demonstrated that ovaries can express tumor necrosis factor (TNF) receptors, and is sensitive to the TNF-α-mediated cell apoptosis pathway [22]. In order to further investigate whether TNF-α participates in chemotherapy-induced apoptosis, we also evaluated the expression of TNF-α in the injured ovaries. TNF-α mRNA was significantly increased in the ovaries of chemoablated mice as compared to controls (Fig. 2B, *p* < 0.05). These results showed that chemotherapy drugs induce GC apoptosis, and increase inflammation within ovarian tissue.

### Grafted hAECs reduce chemotherapy-induced inflammation in ovarian tissue

**Fig. 3:**
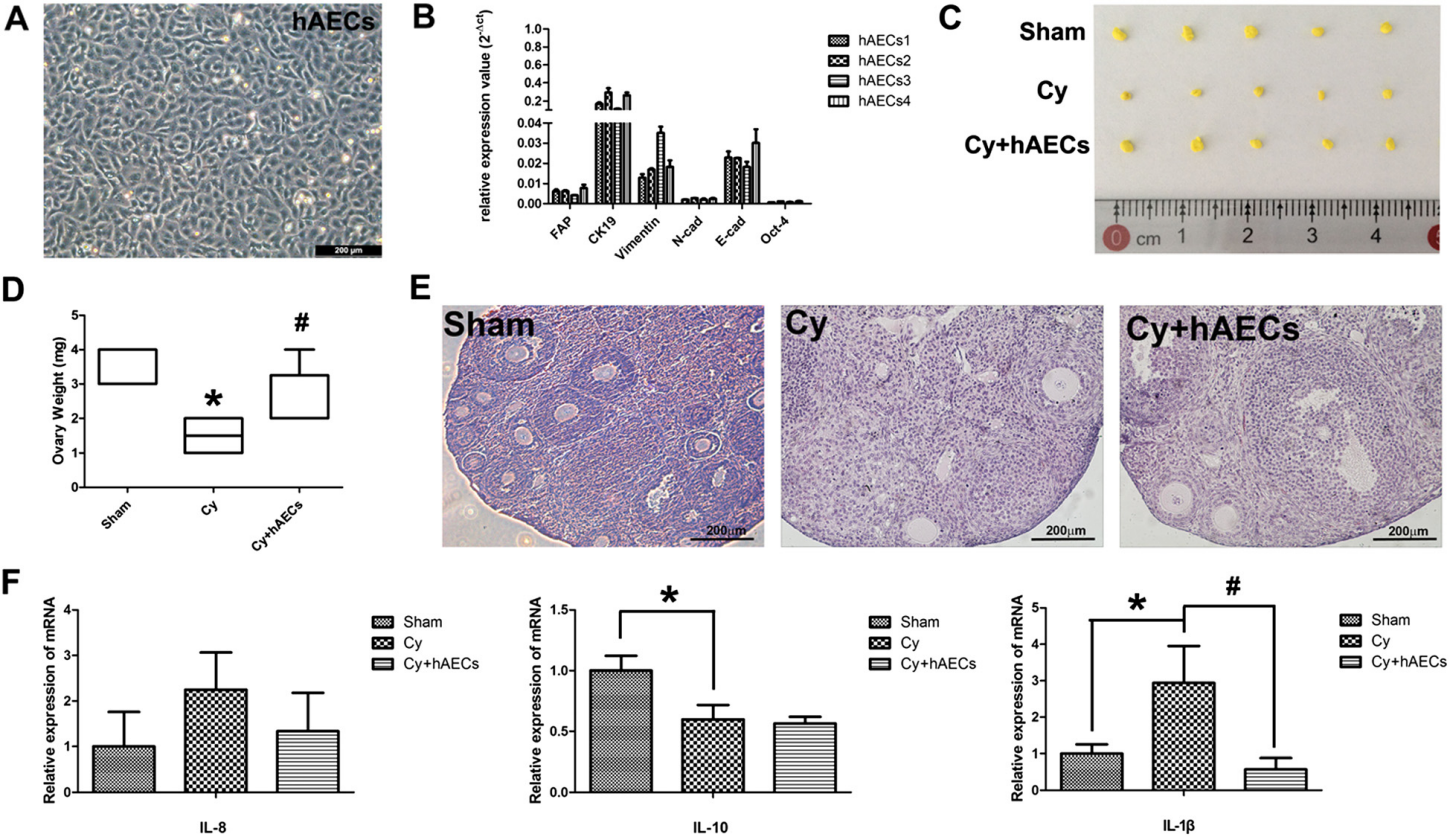
hAEC transplantation reduced chemotherapy-induced inflammation in ovarian tissue. **A** Section of dissected hAECs illustrated a cobble stone-like epithelial cell morphology. **B** Relative expression of mRNA levels detected by real-time PCR in cultured hAECs. **C** Image of fixed ovarian tissue in different groups at 7 days after hAEC transplantation. **D** Bar graph demonstrated the ovarian weights in different groups. **E** H&E staining results displaying ovarian morphology in different groups at 7 days after hAEC transplantation. **F** Relative expression of inflammatory cytokine mRNA levels in injured ovarian tissue analyzed by real-time PCR at 7 days after hAEC transplantation. Data represent means ± SEM; *p < 0.05 versus Sham, #*p* < 0.05 versus Cy. Scale bar = 200 μm in (**E**). *Cy* chemoablated group (n = 6), *Cy + hAECs* hAEC-treated group (n = 6), *Sham* sham group (n = 4)

To eluciate the impact of hAECs on chemotherapy-induced inflammation, we separated hAECs from the fresh amnion of placenta tissue in vitro. Under light microscopy, the primary hAECs appeared as cobble stone-like epithelial cells (Fig. 3A). Real-time PCR demonstrated that cultured hAECs expressed high mRNA levels of Cytokeratin 19 (CK19), Vimentin, E-cadherin (E-cad) and Octamer-binding transcription factor (Oct-4), indicating that hAECs have the character of epithelia cells and stem cells (Fig. 3B). Although hAEC transplantation had no significant effect on the body weight of mice, ovarian weight in the hAEC-treated group was higher than those of the chemoablated group at 7 days (Fig. 3C and D). Moreover, hAEC-treated mice displayed more follicles than chemoablated mice at 7 days after hAEC transplantation (Fig. 3E). Interleukin (IL)-1ß, a pro-inflammatory cytokine, was significantly increased (by real-time PCR analysis) following chemotherapy, while the anti-inflammatory cytokine (IL-10) was significantly reduced in injured ovarian tissue. However, the increase in IL-1ß expression was inhibited in the hAEC-treated group. These results indicate that grafted hAECs may partially inhibit the chemotherapy-induced inflammatory response to achieve the goal of reduced of ovarian injury.

### Grafted hAECs inhibit TNF-α-mediated cell apoptosis in chemoablated mice

**Fig. 4:**
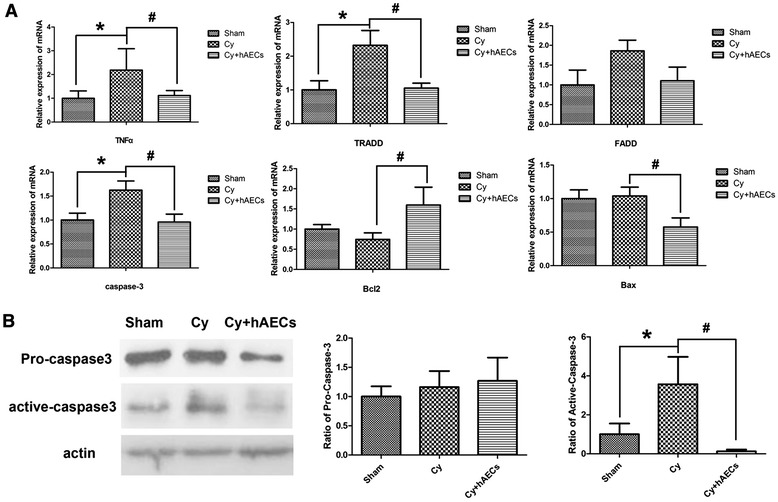
hAEC transplantation inhibited chemotherapy-induced apoptosis in ovaries. **A** Relative mRNA expression of TNF-α, TRADD, FADD, caspase-3, Bcl2 and Bax was detected by real-time PCR in ovarian tissue at 7 days after hAECs transplantation. **B** Western blot results and corresponding densitometry analysis demonstrated that chemotherapy significantly increased activated caspase-3 protein expression at 7 days after hAEC transplantation. A significant reduction of activated caspase-3 protein expression in the hAEC-treated group compared with the chemoablated group. Data are mean ± SEM; *p < 0.05 versus Sham; #*p* < 0.05 versus Cy. *Cy* chemoablated group (n = 6 in **A**, n = 4 in **B**), *Cy + hAECs* hAEC-treated group (n = 6 in **A**, n = 4 in **B**), *Sham* sham group (n = 4 in both panels)

TNF-α is a cytokine effector that not only induces an inflammatory response but also regulates cell proliferation and apoptosis through interactions with different receptors [23]. In the current investigation, chemotherapy increased TNF-α mRNA levels in the ovarian tissue of chemoablated mice (Fig. 2B). We evaluated whether hAEC transplantation could inhibit the activation of key genes of TNF-α-mediated apoptosis pathways such as Fas-associated death domain (FADD), TNFR1-associated death domain protein (TRADD) and caspase-3 in ovarian tissue. Real-time PCR results showed that the mRNA level of TNF-α, TRADD and caspase-3 was higher in ovaries of the chemoablated group than those of the sham group. However, following hAEC transplantation, a significant reduction in the mRNA level of TNF-α, TRADD and caspase-3 was observed in the chemoablated ovaries (Fig. 4A, *p* < 0.05). Furthermore, we observed a significant increase in the mRNA of the antiapoptotic gene, Bcl2, with a concomitant decrease in the expression of the proapoptotic gene, Bax, in ovaries of the hAEC-treated group (Fig. 4A, *p* < 0.05). The protein expression of active caspase-3 in injured ovarian tissue was evaluated by Western blot, revealing increased protein expression following chemotherapy; hAEC transplantation inhibited this process (Fig. 4B, *p* < 0.05). Therefore, these results demonstrate that grafted hAECs inhibit chemotherapy-induced cell apoptosis mediated by TNF-α within the ovaries of these mice.

### Grafted hAECs increase the number of cumulus oocyte complexes in chemoablated mice

**Fig. 5:**
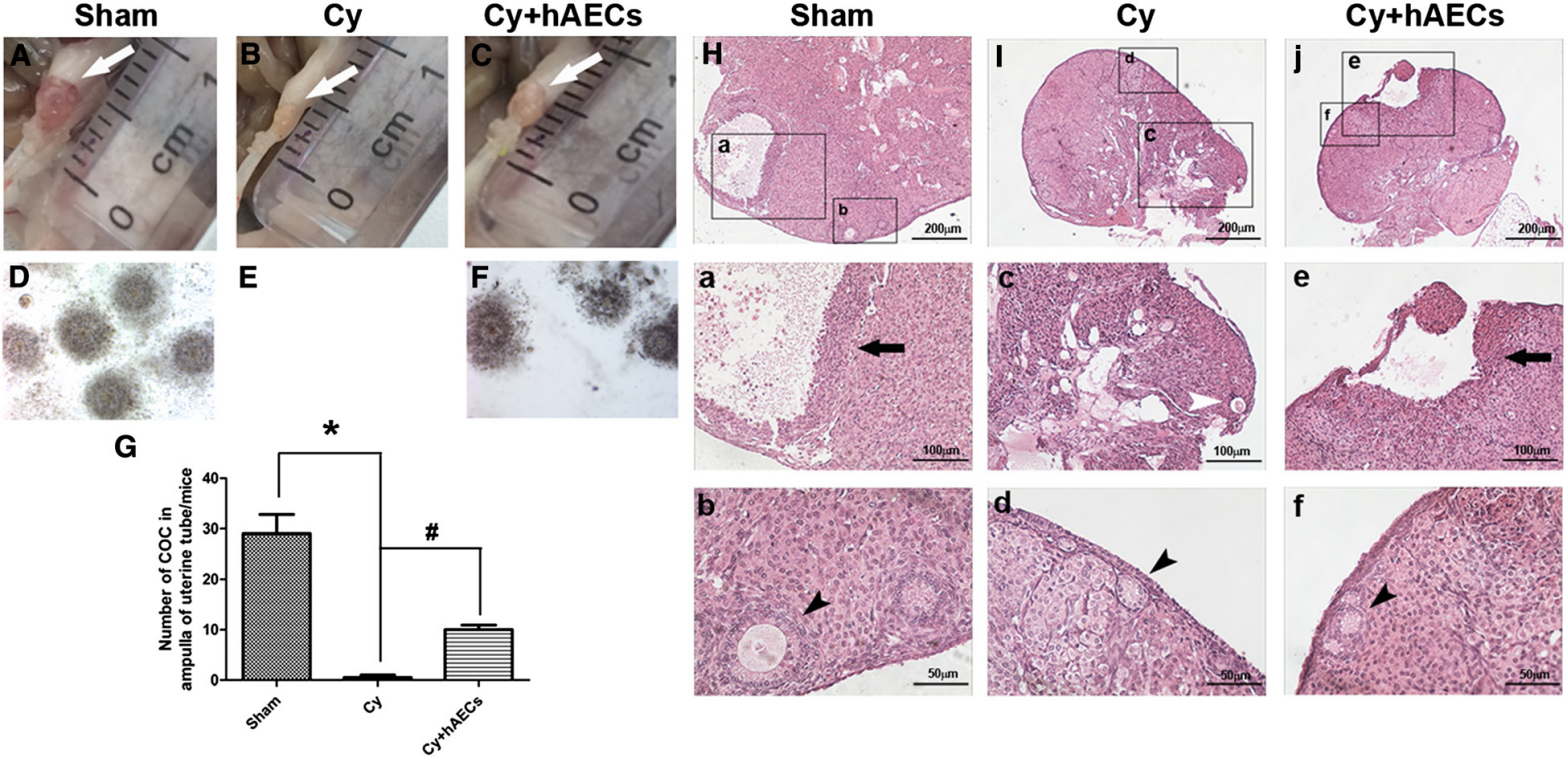
hAEC transplantation increased the number of cumulus oocyte complexes (COC) in chemoablated mice. **A**–**C** Images of ovarian morphology followed superovulation in different groups 1 month after hAEC transplantation (*white arrows*). **D**–**F** The morphology of COC was observed under microscopy. **G** Bar graph representing the counting of COC in different groups. **H**–**J** H&E staining of ovaries followed superovulation was used to observed follicle development. Enlargements of outlined areas are shown in panels *a*–*f*. Mature follicles are indicated by the *black arrow* (*a* and *e*). Primary follicles are indicated by the *black arrowhead* (*b*, *d* and *f*). Follicular atresia is indicated by the *white arrowhead* (*c*). Data are mean ± SEM; *p < 0.05 versus Sham; #*p* < 0.05 versus Cy. Scale bars = 200 μm (**H**–**J**), 100 μm (*a*, *c* and *e*), 50 μm (*b*, *d* and *f*). *Cy* chemoablated group (n = 4), *Cy + hAECs* hAEC-treated group (n = 4), *Sham* sham group (n = 4)

To observe the effect of hAEC transplantation on the process of follicle development, we carried out superovulation in mice at 1 month after hAEC transplantation. Mice were superovulated via a single intraperitoneal injection of gonadotropins. Images showed that the ovaries of mice in the chemoablated group followed superovulation were still atrophic following superovulation as compared to sham and hAEC-treated groups (Fig. 5A–C). Massive COC were acquired from the ampulla portion of the oviduct in the sham group (29 ± 4.397, n = 4). Almost no COC were observed in the chemoablated mice. However, grafted hAECs increased COC (10 ± 1.054, n = 4) compared to the chemoablated group (Fig. 5d–g, *p* < 0.05). Mature follicles could be observed in ovaries of mice in the sham and hAEC-treated groups (Fig. 5*a* and *e*, respectively). Conversely, the chemoablated group only demonstrated follicular atresia with complete absence of mature follicle formation (Fig. 5c). Interestingly, healthy primary follicles could be observed in ovaries followed superovulation in each of groups (Fig. 5*b*, *d* and *f*). These results suggested that hAEC transplantation could rescue the function of secondary follicles and further promote maturation of follicles in chemoablated mice.

### hAEC transplantation promotes ovarian function recovery of chemoablated mice

**Fig. 6:**
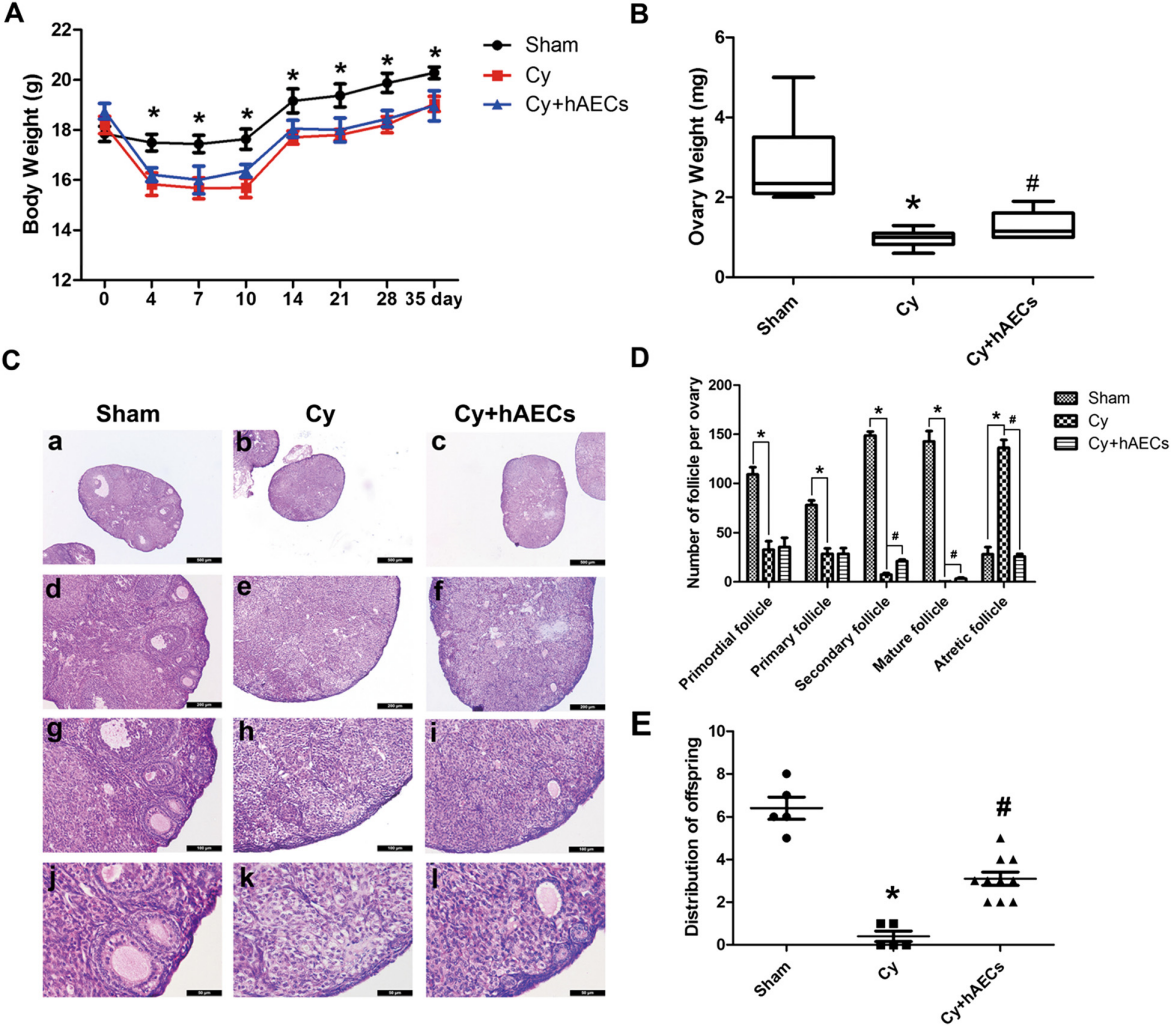
hAEC transplantation increased the number of ovarian follicles and restored the fertility of chemoablated mice. **A** Bar graph illustrating body weight of experimental and control groups following hAEC transplantation. **B** The ovarian weight in different groups at 28 days after hAEC transplantation. **C** Histological analysis of ovaries in different groups at 28 days after hAEC transplantation. **D** The number of different stage follicles counted at 28 days after hAEC transplantation. **E** Bar graph representing the number of pups per pregnancy at the end of mating. Data are mean ± SEM; *p < 0.05 versus Sham; #*p* < 0.05 versus Cy. Scale bars in **c** = 500 μm (*a–c*), 200 μm (*d–f*), 100 μm (*g–i*), 50 μm (*j–l*). *Cy* chemoablated group (n = 6; n = 10 in **E**), *Cy + hAECs* hAEC-treated group (n = 6; n = 10 in **E**), *Sham* sham group (n = 6; n = 5 in **E**)

To investigate the long-term effects of hAEC transplantation on ovarian function, we recorded changes in body weight at various time points following hAEC transplantation. The body weight and ovarian weight of mice in the chemoablated group were significantly lower than those in the sham group. While hAEC transplantation had no obvious effect on body weight (Fig. 6A), the ovarian weight in the hAEC-treated group was significantly higher than that in the chemoablated group at 28 days following transplantation (Fig. 6B, *p* < 0.05). Histologic evaluation indicated that ovaries were severely atrophic, and the population of each stage of follicles was significantly decreased in chemoablated mice (Fig. 6C). hAEC transplantation greatly increased the number of healthy follicles, especially secondary and mature follicles, and attenuated the number of follicular atresia compared to the chemoablated group (Fig. 6C and D, *p* < 0.05).

To assess the impact of hAEC treatment on mouse fertility, female mice were mated with normal males for 1 month, and the total number of pups per pregnancy was counted. During the three mating sessions, mice in the chemoablated group lost reproductive ability compared to mice in the sham group. Conversely, the total number of pups born in the hAEC-treated group was greater than those in the chemoablated group (Fig. 6E, *p* < 0.05). These results demonstrate that hAEC transplantation can reduce chemotherapy-induced ovarian injury and restore the recovery of ovary function.

## Discussion

As the incidence of POF/POI continues to increase, there is a growing need to identify novel interventions and treatment strategies. In the current study, we utilized a mouse model to mimic the clinical features of POF/POI, including the progressive loss of follicles and the decline in fertility. The results of this study demonstrate that hAEC transplantation post-chemotherapy mitigates chemotherapy-induced ovarian injury and restores the fertility of chemoablated mice. These observations suggest that hAEC transplantation may represent a future treatment strategy for individuals suffering from POF/POI.

Chemotherapy with alkylating agents, while critically important to cancer therapy, can result in unintended and severe consequences, such as female infertility. Mechanistically, infertility results from a biological cascade effect, which includes progressive loss of follicles and extensive apoptosis of GCs [24]. GCs are an essential component of the ovarian microenvironment and play a key role in regulating reproductive ovarian physiology, including ovulation and luteal regression. Previous research has revealed that chemotherapy drugs accelerate follicular atresia, and this process is characterized by GC apoptosis [25, 26]. As supported by previous reports, we observed a significant reduction in the weight of mice and the number of follicles in ovarian tissue following administration of chemotherapy (Fig. 1). Prior evidence had demonstrated that docetaxel induces moderate ovarian toxicity in mice, primarily affecting GCs of early growing follicles [27]. Consistent with these findings, we also observed the chemotherapy-induced destruction of GCs in the developing follicle, especially in the secondary follicle (Fig. 2). Thus, the targeting of antiapoptotic activity may be a therapeutic strategy to protect the ovary against chemotherapy-induced injury.

TNF-α is an important regulatory cytokine, which not only regulates the immune response but also influences cell differentiation, survival and apoptosis. Two disparate signaling pathways can be induced by TNF-α depending on the specific ligand–receptor interaction. The extrinsic apoptosis pathway is activated when the ligand of TNF-α binds to its death receptors (tumor necrosis factor receptor 1 (TNFR1)) on the cell membrane [23, 28]. Researchers have demonstrated that the ovaries of mice express both TNF receptors and are sensitive to TNF-α-mediated death pathway [22]. Furthermore, in post-chemotherapy cancer patients, increased TNF-α is observed in addition to enhanced TNF-related apoptosis [29]. Moreover, intraovarian transplantation of primordial follicles is unable to rescue chemotherapy-induced ovarian injury [30]. Thus, to restore the ovarian function, it is necessary to inhibit apoptosis of GCs and ameliorate the ovarian microenvironment of chemoablated mice.

hAECs have been shown to be broadly multipotent and nontumorigenic, thereby representing an attractive source for stem cell therapy. Many studies indicate that the release of cellular growth factor from transplanted stem cells stimulates tissue regeneration, coupled with cells that may undergo a transdifferentiation to specific tissue cells [31, 32]. In a previous study, we demonstrated that grafted hAECs could migrate into injured ovarian tissue and differentiated into GCs around oocytes [18]. In the current investigation, we further characterized the effect of hAECs on chemotherapy-induced apoptosis and inflammation in ovarian tissue. Our results demonstrate that the mRNA levels of pro-inflammatory cytokines (such as TNF-α, IL-8 and IL-1ß) are significantly upregulated, and their elevation was observed in conjunction with the increase in follicular atresia in the ovaries of chemoablated mice. Increased recruitment of FADD and TRADD to the TNF-α-induced cell death signaling pathway was shown in mice exposed to chemotherapy drugs. Additionally, administration of chemotherapy significantly increased active caspase-3 protein expression compared with the sham group (Fig. 4). In contrast, hAEC transplantation significantly reduced the mRNA level of FADD, TRADD and caspase-3 in injured ovarian tissue of chemoablated mice. Thus it can be seen that hAEC injection could effectively alleviate the chemotherapy-induced inflammatory reaction in ovarian tissue. A previous study demonstrated that human amnion epithelium stains positively for IL-4 by immunohistochemistry, and these cells can suppress the production of TNF-α, IL-1 and IL-6 by activated monocytes [33]. Thus, hAEC transplantation may inhibit TNF-α-mediated apoptosis and reduced inflammation in chemotherapy-induced ovarian injury.

Notably, the beneficial effects of hAEC administration may rely on genetic and environmental factors. For instance, hAECs failed to rescue bleomycin-induced lung injury in a mouse strain with a defective macrophage function [34]. However, it was demonstrated that hAECs could improve lung repair by directly modulating macrophage recruitment and polarization [14]. Thus, macrophages are likely a critical component required for effective hAEC transplantation therapy for tissue injury. Whether hAEC transplantation in chemotherapy-treated mice could restore ovary function through modulating the immunologic function of recipient mice would be worth further exploring. Additionally, transplanted hAECs may also secret anti-inflammatory factors via a paracrine pathway, and promoting the secretions of growth factors may lead to a local microenvironment more conducive for follicle growth and development. Recently, research have reported that cultured human amnion secreted various growth factors, such as fibroblast growth factor-6, neurotrophin-4, vascular endothelial growth factor receptor-3, macrophage colony-stimulating factor receptor and heterodimer of platelet derived growth factors AB, which may have effects within regenerating damaged tissue [35]. While the current study describes an interesting phenomenon and hints at a partial mechanism for hAEC-induced ovarian regeneration after chemotherapy-induced POF, there is still much to be understood about the effect of cytokines secreted by hAEC on GCs and follicular development of chemoablated mice using the co-culture system.

## Conclusion

The present study provides important evidence that hAEC transplantation could effectively improve ovarian function by inhibiting cell apoptosis and reducing inflammation in injured ovarian tissue of chemoablated mice. hAEC transplantation could serve as a potential and promising new strategy for the management of POF/POI in female cancer survivors.

## Additional file

Additional file 1: Table S1. PCR primers used to detect gene expression in ovaries of mice (m) and human amniotic epithelial cells (h). (DOCX 17 kb)

## Abbreviations

ANOVA: Analysis of variance
CK19: Cytokeratin 19
COC: Cumulus oocyte complexes
Cy: Chemoablated group
E-Cad: E-cadherin
FADD: Fas-associated death domain
FBS: Fetal bovine serum
GC: granulosa cell
H&E: Hematoxylin-eosin
hAEC: Human amniotic epithelial cell
hCG: human chorionic gonadotropin
IL: Interleukin
LSD: Least significant difference
Oct-4: Octamer-binding transcription factor 4
PBS: Phosphate-buffered saline
PMSG: Pregnant mare serum gonadotropin
POF/POI: Premature ovarian failure and insufficiency
PVDF: polyvinyldifluoridine
SEM: Standard error of the mean
SSS: Serum substitute supplement
TNF: Tumor necrosis factor
TNFR1: Tumor necrosis factor receptor 1
TRADD: TNFR1-associated death domain
