# Supplementary material for: Human amniotic epithelial cells inhibit granulosa cell apoptosis induced by chemotherapy and restore the fertility
Source: Stem Cell Res Ther. 2015 Aug 25;6(1):152. doi: 10.1186/s13287-015-0148-4 (PMC4549019; doi:10.1186/s13287-015-0148-4)
Supplement: Additional file 1: Table S1. — PCR primers used to detect gene expression in ovaries of mice (m) and human amniotic epithelial cells (h). (DOCX 17 kb) [file 13287_2015_148_MOESM1_ESM.docx]

Table 1. PCR primers used to detect gene expression in ovaries of mice (m) and human amniotic epithelial cells (h).

| **Gene symbol** | **Primer sequence** | | | **Fragment**  **size (bp)** |
| --- | --- | --- | --- | --- |
| mBcl2 | Forward: | TTGTAATTCATCTGCCGCCG |  | 179 |
|  | Reverse: | AATGAATCGGGAGTTGGGGT |  |  |
| mBax | Forward: | GAGACACCTGAGCTGACCTT |  | 203 |
|  | Reverse: | CCCCAGTTGAAGTTGCCATC |  |  |
| mTNFα | Forward: | CACCATGAGCACAGAAAGCA |  | 154 |
|  | Reverse: | TAGACAGAAGAGCGTGGTGG |  |  |
| mIL-8 | Forward: | CTCCTGCTGGCTGTCCTTAA |  | 152 |
|  | Reverse: | CTGTTGCAGTAAATGGTCTCGA |  |  |
| mIL-10 | Forward: | TAAGGCTGGCCACACTTGAG |  | 209 |
|  | Reverse: | GTTTTCAGGGATGAAGCGGC |  |  |
| mIL-1β | Forward: | TGCCACCTTTTGACAGTGATG |  | 220 |
|  | Reverse: | AAGGTCCACGGGAAAGACAC |  |  |
| mTRADD | Forward: | GAGCTGCTGGAGTGCAACTA |  | 129 |
|  | Reverse: | GGTCCGGGTACTTAGAGGGT |  |  |
| mFADD | Forward: | TGAGAAGAAGAACGCCTCGG |  | 181 |
|  | Reverse: | GGAGCTGTAGGCTTGTCAGG |  |  |
| mCaspase-3 | Forward: | AGTGACCATGGAGAACAACAA |  | 107 |
|  | Reverse: | CCAGATAGATCCCAGAGTCCAC |  |  |
| mGAPDH  hFAP  hCK19  hVimentin  hN-cad  hE-cad  hOct-4 | Forward: | CCAATGTGTCCGTCGTGGATCT | | 149  215  211  100  120  131  224 |
|  | Reverse:  Forward:  Reverse:  Forward:  Reverse:  Forward:  Reverse:  Forward:  Reverse:  Forward:  Reverse:  Forward:  Reverse: | GTTGAAGTCGCAGGAGACAACC  ATGAGCTTCCTCGTCCAATTCA  AGACCACCAGAGAGCATATTTTG  TTTGAGACGGAACAGGCTCT  AATCCACCTCCACACTGACC  AGGCAAAGCAGGAGTCCACTGA ATCTGGCGTTCCAGGGACTCAT  TTTGATGGAGGTCTCCTAACACC  ACGTTTAACACGTTGGAAATGTG  GCCTCCTGAAAAGAGAGTGGAAG  TGGCAGTGTCTCTCCAAATCCG  GGCCCGAAAGAGAAAGCGAACC ACCCAGCAGCCTCAAAATCCTCTC |  |  |
